# Cervicothoracic ventral-dorsal rhizotomy for treatment of brachial hypertonia in cerebral palsy

**DOI:** 10.1007/s00381-024-06479-5

**Published:** 2024-07-03

**Authors:** Sunny Abdelmageed, Mahalia Dalmage, James M. Mossner, Robin Trierweiler, Tim Krater, Jeffrey S. Raskin

**Affiliations:** 1 Division of Pediatric Neurosurgery, Department of Surgery, Ann and Robert H. Lurie Children’s Hospital, 211 E Chicago Ave Ste 1150, Chicago, IL 60611 USA; 2 Department of Neurosurgery, Northwestern University Feinberg School of Medicine, Chicago, IL USA; 3 Department of Neurosurgery, University of Chicago Pritzker School of Medicine, Chicago, IL USA; 4 Nuvasive Clinical Services, Columbia, MD USA; 5 Shirley Ryan Ability Lab, Chicago, IL USA

**Keywords:** Brachial hypertonia, Cerebral palsy, Cervical rhizotomy, Combined rhizotomy, Mixed hypertonia

## Abstract

**Purpose:**

Cervicothoracic ventral-dorsal rhizotomy (VDR) is a potential treatment of medically refractory hypertonia in patients who are not candidates for intrathecal baclofen, particularly in cases of severe upper limb hypertonia with limited to no function. A longitudinal cohort was identified to highlight our institutional safety and efficacy using cervicothoracic VDR for the treatment of hypertonia.

**Methods:**

Retrospective data analysis was performed for patients that underwent non-selective cervicothoracic VDR between 2022 and 2023. Non-modifiable risk factors, clinical variables, and operative characteristics were collected.

**Results:**

Six patients (three female) were included. Four patients underwent a bilateral C6-T1 VDR, one patient underwent a left C7-T1 VDR, and another underwent a left C6-T1 VDR. Three patients had quadriplegic mixed hypertonia, one patient had quadriplegic spasticity, one patient had triplegic mixed hypertonia, and one patient had mixed hemiplegic hypertonia. The mean difference of proximal upper extremity modified Ashworth scale (mAS) was − 1.4 ± 0.55 (*p* = 0.002), and − 2.2 ± 0.45 (*p* < 0.001) for the distal upper extremity. Both patients with independence noted quality of life improvements as well as increased ease with dressing and orthotics fits. Caregivers for the remaining four patients noted improvements in caregiving provision, mainly in dressing, orthotics fit, and ease when transferring.

**Conclusion:**

Cervicothoracic VDR is safe and provides tone control and quality of life improvements in short-term follow-up. It can be considered for the treatment of refractory hypertonia. Larger multicenter studies with longer follow-up are necessary to further determine safety along with long-term functional benefits in these patients.

## Introduction

Hypertonia is a form of hyperkinetic movement disorder further defined by dystonia, spasticity, or a mixture of these pathologies. It can be classified as general, focal, segmental, or limb specific. Hypertonia in cerebral palsy (CP) occurs secondary to altered cerebral development or injury, including traumatic injury, non-traumatic injury, hypoxic ischemic encephalopathy, central nervous system (CNS) infection, stroke, or genetics [1–3].

Hypertonia is initially treated with medical interventions like bracing, physiotherapy, serial casting, injections with botulinum toxin or phenol, and antispasmodic medications [4–6]. Medically refractory cases may require neurosurgical interventions. Classic neurosurgical interventions to treat generalized hypertonia include thalamotomy or pallidotomy, deep brain stimulation (DBS), or intrathecal baclofen pump therapy (ITBP) [7–10].For patients with limb-specific hypertonia, these neurosurgical interventions may be too broad, and a more targeted approach such as peripheral rhizotomy may be more beneficial.

The first account of peripheral rhizotomy, selective dorsal rhizotomy (SDR), was in 1888 when Dr. Charles Dana and Dr. Robert Abbe conceived and performed a dorsal rhizotomy to alleviate pain and spasticity in the arm of a patient [11].Throughout the twentieth century, SDR gained popularity and was improved upon to reduce complications and increase nerve identification reliability [12, 13]. Today, rhizotomies can be performed as ventral, dorsal, or combined from the cervical to sacral spine with a selective or non-selective (> 50% root sectioning) approach.

Combined ventral-dorsal rhizotomies (VDR) can be a reasonable choice for patients with non-generalized conditions because it can address their spasticity via dorsal rhizotomy and their dystonia through ventral rhizotomy. Though lumbosacral rhizotomy is more popular, cervical rhizotomy has shown success in the treatment of upper limb spasticity, traumatic hypertonia, and torticollis [14–18]. Albright and Tyler-Kabara describe the first use of cervicothoracic VDR in one patient with improvement in tone control [18]. Despite these promising indications, there remains a dearth of studies on the role of cervicothoracic VDR for management of brachial hypertonia in children and young adults with CP.

We identified a retrospective cohort of patients undergoing non-selective cervicothoracic VDR for the treatment of brachial hypertonia. This study aims to investigate the role of non-selective cervicothoracic VDR as a palliative tone management for children and young adults with medically refractory brachial hypertonia.

## Methods

### Patient selection

Approval by the Ann & Robert H. Lurie Children’s Hospital of Chicago (LCH) Institutional Review Board (IRB: 2023–6437) was obtained prior to initiation of this study. A retrospective single-center chart review was conducted on patients who underwent a cervical VDR between January 2022 and November 2023 and were selected from a pre-established surgical database maintained by the treating physician JSR.

### Demographic factors

Demographic factors such as age, prematurity less than 37 weeks gestational age, scoliosis, and procedures performed for comorbid conditions were collected. Procedures included gastrostomy-tube placement, tracheostomy, ITBP placement, and prior spinal fusion. We also collected clinical data related to their condition including etiology of hypertonia and gross motor function classification scale scores (GMFCS).

### Surgical procedure

**Fig. 1:**
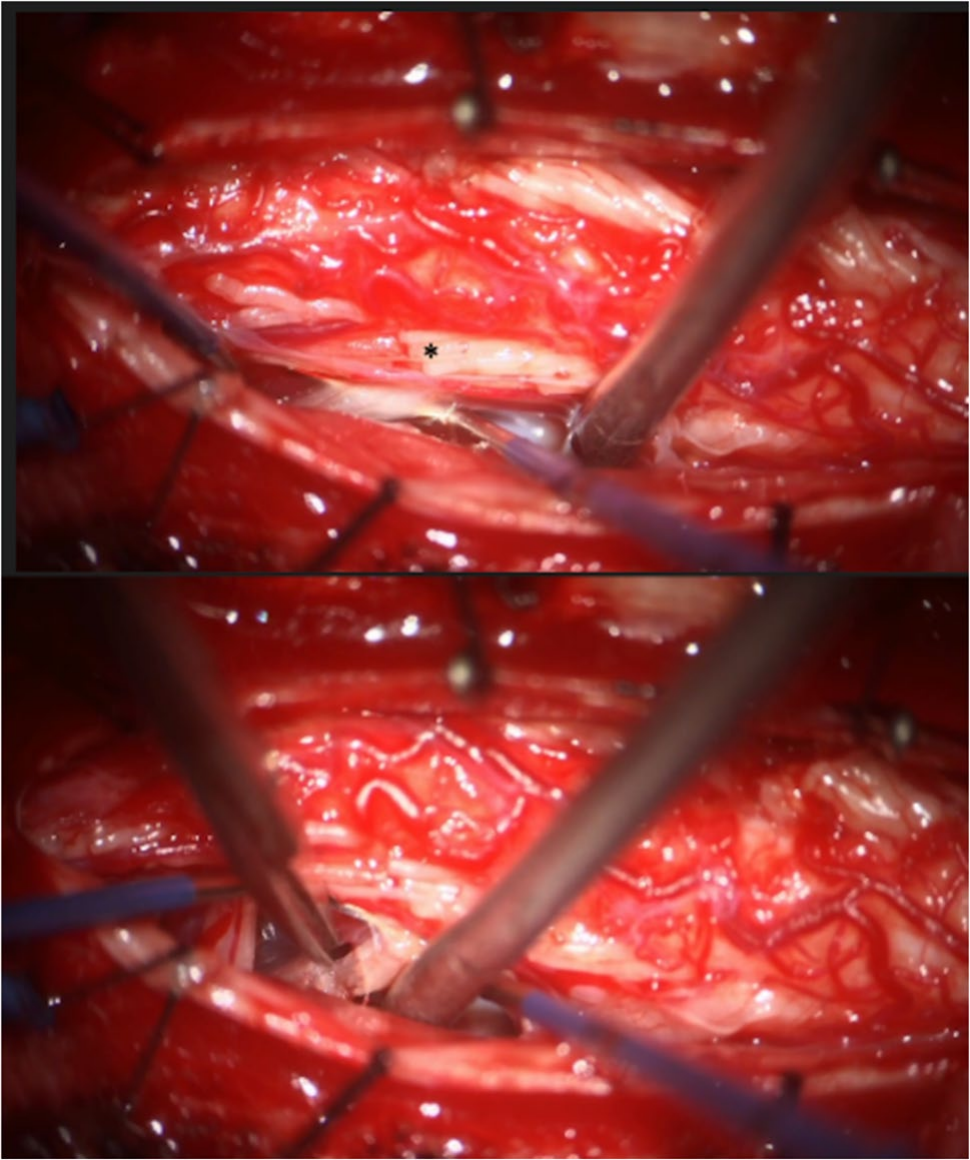
Intraoperative cervicothoracic ventral-dorsal rhizotomy photographs. Patient head is oriented to the right, dura has been opened via midline durotomy. Gillette nerve hooks are used to dissect the arachnoidal root sleeve and then stimulate to identify the appropriate myotome. Top demonstrates the ventral root on the nerve hook after partial dorsal root sectioning (*) via a window created by deflection of the uncut dorsal rootlets within the right lateral recess. Bottom demonstrates ventral nerve rootlet sectioning in the right lateral recess

Cervicothoracic VDR is performed prone using triggered electromyography (EMG). All surgeries are performed following typical antibiosis and surgical pause under general anesthesia. A midline incision overlying C5-T1 is performed; levels are confirmed with fluoroscopy. Paraspinal muscle dissection and laminoplasty are performed for dural access. The dura is opened, and the arachnoidal investment of the cervical spinal cord is opened using microneurosurgical instruments. The most cephalad mixed nerve root is gathered using the Gillette nerve hooks and stimulated with tetanic stimulation using 50 Hz to note activation of the C6 Myotome. Then, the dorsal root is independently gathered, stimulated to threshold, and 80–90% is sectioned using microscissors (Fig. 1).

This process is repeated for the ventral root and then continued sequentially in a caudal direction until T1 is sectioned. Positioning adjustments can assist in accessing the ventral roots. Sometimes, it is feasible to section the ventral rootlets through a new corridor created by sectioning the dorsal rootlets. If performing bilaterally, the process is repeated on the other side. Following hemostasis, the dura is repaired, a laminoplasty performed, and layered muscle and skin closure in the usual fashion. Although roots are non-selectively sectioned, intraoperative EMG is necessary to confirm the spinal level. Additional irrigation is only added to the field if CSF has egressed ensuring that EMG monitoring remains accurate. Our specific cervicothoracic VDR technique has been described in further detail with operative video previously [19].

At our institution, ideal candidates for the procedure have medically refractory hypertonia, are at their developmental maximum, may be contraindicated for other neurosurgical interventions, and are seeking a palliative option rather than a gain-of-function treatment.

### Outcome measures and data analysis

Surgical characteristics including hospital length of stay, operative time, estimated blood loss, nerve roots sectioned, and percentage of those roots sectioned were collected. Mean operative time was calculated using patients that underwent cervicothoracic VDR only; patients that underwent concurrent procedures were excluded from this calculation.

Primary outcome measures included tone control assessed quantitatively (pre-operative and post-operative modified Ashworth scale (mAS) or Barry Albright Dystonia Scale (BADS)), and quality of life assessed qualitatively (ease of transfers, ability to fit orthotics, etc.). Quality of life metrics were determined using patient perspective when possible or by caregiver perspective. Functional improvement was assessed in some patients using the Manual Ability Classification System (MACS). Secondary outcome measures included postoperative complications as a marker for safety. Postoperative complications included wound dehiscence, infection, pneumonia, and respiratory depression—measured by desaturations necessitating respiratory support.

Patients underwent clinical scale testing preoperatively and at 3 months postoperatively using the mAS, BADS or MACS by a licensed pediatric physical medicine and rehabilitation specialist through the Shirley Ryan AbilityLab. The mAS ranges from 0 to 4 for a single muscle or joint being rapidly moved through a given range, where 0 is no increase in muscle tone, and 4 is a muscle group rigid in flexion or extension [20]. We separated the upper extremity mAS scores into proximal and distal scores to discern if there was any difference in efficacy related to proximity to the surgical site.

Mean difference was calculated by subtracting the baseline score from the postoperative, then obtaining the mean of differences. A paired, one-tailed Student’s T-test was used to compare pre- and post-operative mAS scores with *p* ≤ 0.05 considered significant. All statistical analysis were completed using RStudio (RStudio Team, 2023).

## Results

Our study yielded a total of six patients (three female). Patient demographics are described in Table 1.

**Table 1:** Demographics and baseline clinical information

Case no	Age, sex	Race, ethnicity	Type of hypertonia	CP etiology	GMFCS	G-tube	Scoliosis
1	22, M	Hispanic/Latino	Triplegic, mixed	Post-meningitis hydrocephalus	II	No	No
2	14, M	NHW	Tetraplegic, mixed	Schizencephaly	V	Yes	Yes
3	9, F	NHW, Black	Tetraplegic, mixed	Nonaccidental trauma	V	Yes	Yes
4	15, F	Hispanic/Latino	Tetraplegic, mixed	Microlissencephaly	V	Yes	Yes
5	7, M	Hispanic/Latino	Tetraplegic, spastic	Unknown	V	Yes	Yes
6	34, F	NHW	Hemiplegic, mixed	IVH	I	No	No

*No* number, *GMFCS* gross motor function classification scale, *M* male, *F* female, *NHW* non-Hispanic White, *CP* cerebral palsy, *IVH* intraventricular hemorrhage; *G-tube* gastrostomy tube

Mean age at surgery was 16.8 years (range 7–34). Three patients had quadriplegic mixed hypertonia, one patient had quadriplegic spasticity, one patient had triplegic mixed hypertonia, and one patient had mixed hemiplegic hypertonia. Fifty percent of patients were delivered prematurely; mean gestational age was 34 weeks (range 26–40 weeks). CP etiologies included CNS injury, CNS infection, genetics, and unknown. Four patients had a GMFCS of V, one had a score of II, and one had a score of I. Four patients had a gastrostomy tube and scoliosis, no patients had a tracheostomy tube, ITBP placement, or prior spinal fusion.

Four patients underwent a bilateral C6-T1 VDR, one patient underwent a left C7-T1 rhizotomy, and another underwent a left C6-T1 rhizotomy. Mean operative duration was 239 ± 25.2 min, mean estimated blood loss was 93.3 ± 83.2 mL, and mean length of stay was 7 ± 3.9 days (Table 2).

**Table 2:** Surgical characteristics and perioperative events

Case no	Surgery	Roots cut, %	LOS (days)	Op time (min)	EBL (ml)	Follow-up (days)	Complications
1	Left C7-T1	80	7	252	200	392	---
2	Bilateral C6-T1	80–90	3	552*	200	105	--
3	Bilateral C6-T1	80–90	14	457*	50	118	Transient BiPap support
4	Bilateral C6-T1	80–90	8	491*	50	206	Transient BiPap support
5	Bilateral C6-T1	80–90	--^	210	30	136	--
6	Left C6-T1	50 dorsal 80 ventral	4	239	30	42	--

*pt underwent concurrent lumbosacral VDR

^LOS is not reported because this surgery was performed during a prolonged hospitalization for various issues to help address subsequent tone control*No* number, *LOS* length of stay, *Op* operative, *EBL* estimated blood loss, *min* minutes, *BiPap* bilevel positive airway pressure

All patients followed the post-operative rehabilitative treatment protocol, which included outpatient rehab after hospital discharge, ranging from one to five times a week. Rehab focused on enhancing range of motion, postural alignment, and maximizing strength of functional patterns. Patient goals for physical therapy were case dependent.

**Fig. 2:**
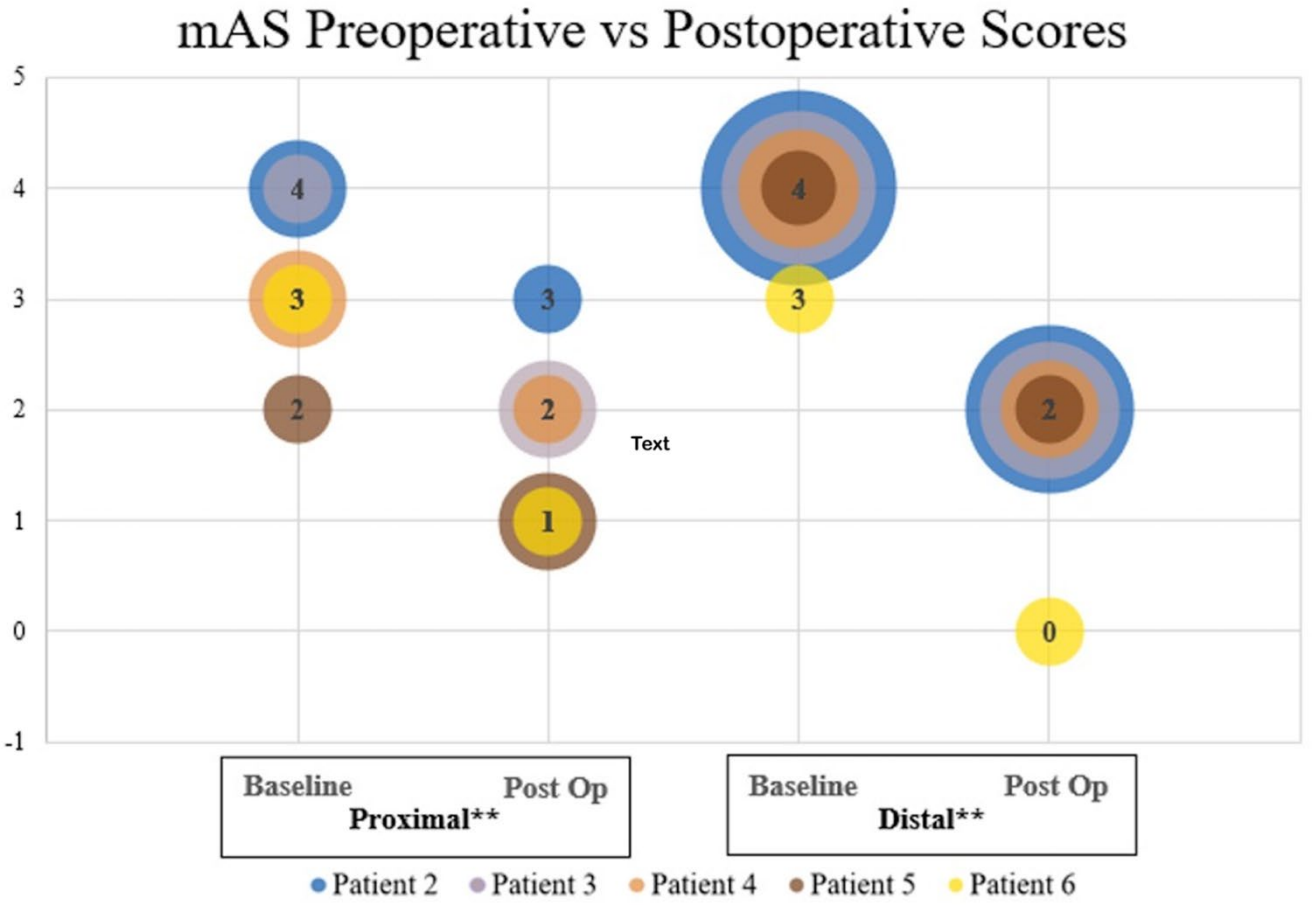
Upper extremity mAS scores preoperative vs. postoperative scores. Bubble plot depicting preoperative and postoperative upper extremity modified Ashworth scale (mAS) scores for patients 2–6. Patient 1 had pure dystonia and therefore did not undergo mAS testing. Both proximal and distal showed significant improvement postoperatively. ** *p* < 0.01, ***p < 0.001

Average follow-up was 191.4 days (range 42–392 days). The mean difference of proximal upper extremity mAS was − 1.4 ± 0.55 (*p* = 0.002) and − 2.2 ± 0.45 (*p* < 0.001) for the distal upper extremity (Fig. 2).

There was not a significant difference between pre- and post-operative mAS in the lower extremities (*p* > 0.05). All patients demonstrated self-reported physical improvements and quality of life improvements, mainly in dressing, orthotics fit, and ease when transferring (Table 3).

**Table 3:** Qualitative outcomes

Case no	Quality of life improvement	Physical improvement*
1	Dressing, orthotics	No significant dystonia, can flex/extend arm. Able to supinate with good control. Able to hold a tray with his left arm. Able to hold controller and play video games better
2	Positioning, transfers	Able to extend arm straight
3	Positioning	Able to open hand, previously fisted
4	Passive dressing, changing, transfers, orthotics	Improved tone
5	Passive dressing, changing, positioning, transfers, orthotics	Improved tone, no clonus, full range of motion
6	Dressing, orthotics	Improved grip Able to close and open hand around ball. Can stack rings
*No* number, *G-tube* gastrostomy tube, *GA* gestational age, *C* cervical, *physical improvements are qualitative and self-reported		

Two patients with existing restrictive lung disease required transient increased BiPAP for 1 and 3 days following surgery. There was no incidence of infection, pneumonia, or wound dehiscence. Two patients had available follow-up at 6 months post-operatively and exhibited sustained tone control and quality of life improvements. Long-term functional outcomes were not available for the remaining four patients.

### Case 1

A 22-year-old man presented to the movement disorders clinic with triplegic mixed hypertonia secondary to CP caused by post-meningitic hydrocephalus. This patient has trialed botulinum toxin injections, multiple antispasmodic medications, and baclofen which was discontinued due to lowered seizure threshold.

On examination, he had a GMFCS of II and preoperative MACS of 5. He had dystonia in his left upper extremity with BADS of 8. His brachial hypertonia was interfering with his ability to complete job requirements and causing significant pain.

A left C7-T1 VDR was performed with 80% of both the ventral and dorsal roots sectioned. There was a significant reduction in tone; his BADS was 6 at 6-months post-operatively and 3 at 1-year post-operative. At 1-year post-operatively, his MACS score was a 3, and he demonstrated improved function in his left arm. He met 100% of his goals which included holding a tray in his left hand while opening a door with his right hand; furthermore, he was able to supinate with good control. He noted improvement in dressing and wearing of orthotics and was able to perform his job successfully.

### Case 6

A 34-year-old woman presented with hemiplegic mixed hypertonia secondary to CP caused by intraventricular hemorrhage. She had trialed multiple antispasmodics and botulinum toxin injections without adequate effect.

On examination, she had a GMFCS of I and a preoperative MACS of 5. She had severe left brachial hypertonia with a mAS of 3 in her proximal and distal left upper extremity. Her hand was rigid in dystonic posture and she was unable to use it.

A left C6-T1 VDR was performed with 50% of dorsal and 80% of ventral roots sectioned. Post-operatively, there was a significant reduction in tone. At 42 days post-operatively, her she demonstrated a proximal mAS of 1 with complete improvement in distal hypertonicity (mAS = 0). She met 100% of her goals which included opening and closing her hand. At 3-months post-operativel, she was able to open and close her hand around a ball and transfer rings on a post. She also demonstrated improved grip due to decreased interference by tone. She was extremely pleased with the result and noted improvement in dressing and orthotics wearing.

## Discussion

Cervicothoracic SDR has demonstrated success in the treatment of upper limb spasticity showcasing notable reductions in tone [17]. Lumbosacral VDR has shown efficacy in the treatment of lower limb mixed hypertonia, yet there remains a paucity of literature on the use of cervicothoracic VDR [18, 21]. We present the largest case series, to date, for the use of cervicothoracic VDR for the treatment of hypertonia in CP.

Albright and Tyler-Kabara utilized cervical VDR in three pediatric patients sectioning an average of 77.83% of the dorsal root and 66.67% of the ventral root [18]. One cervicothoracic VDR was performed VDR sectioning 80% of the dorsal root and 66% of the ventral roots (Table 4).

**Table 4:** Comparison with previous cohort

Manuscript	Type of rhizotomy	Avg extent of dorsal rhizotomy	Avg extent of ventral rhizotomy	mAS MD	Age
Albright and Tyler-Kabara [22]	Bilateral C5-8 (*n* = 3)	77.83%	66.67%	-1.46	8–13
	Bilateral C6-T1 (*n* = 1)	80%	66%	-1.4	7.5
Current series	Bilateral C6-T1 (*n* = 4)	85%	85%	-1.75	7–15
	Left C7-T1 (*n* = 1)	80%	80%	--	22
	Left C6-T1 (*n* = 1)	50%	80%	-2.5	34
--, mAS not reported because this patient had mainly dystonia					

The rationale behind the inclusion of T1 in only one patient in their series is unclear to us; however, it may represent differences in hypertonia localization. Our cohort demonstrated significant distal hypertonia including abnormal hand posturing. The inclusion of T1 was particularly important due to its role in the innervation of the intrinsic hand muscles and its capacity to facilitate hand-opening.

Like the prior study, we report success in decreasing mAS scores and reducing muscle rigidity during our three-month follow-up with minimal complications [18]. Selective sectioning is not necessary as this procedure is intended for palliative tone control rather than gain-of-function. We consistently non-selectively sectioned 80–90% of the ventral root and 50–80% of the dorsal root, the highest reported in the literature, and each patient showed improvement in postoperative mAS score. It is theorized for achieving an effective response; patients with dystonia require a higher percentage of nerve root sectioning; thus, the benefit of increased root lesioning is the decreased likelihood of needing a subsequent revision surgery [18]. However, increased sectioning also contributes to the permanence of the procedure and may prevent patients from potential gain-of-function treatments in the future. There was a greater improvement in distal upper extremity mAS score compared with the proximal upper extremity which is likely explained by the greater baseline mAS score in the distal limb, but could represent differences due to ease of mAS administration in the distal limb.

These reductions in mAS score are mirrored by the improved quality-of-life (QoL) improvements reported by patients and caregivers. Patients with a GMFCS V score (cases 2–5) had quadriplegic hypertonia and needed significant assistance with nearly all activities of living. For these patients, caregivers reported increased ease in positioning and caretaking following cervicothoracic VDR. These QoL improvements underscore the utility of cervicothoracic VDR in severe generalized hypertonia for palliative purposes.

The patients with GMFCS scores II and I (cases 1 and 6, respectively), who were relatively independent, reported improved ease in dressing and orthotics due to decreased muscle tone. These findings illustrate the success of cervicothoracic VDR in instances characterized by predominant brachial hypertonia.

### Indications and considerations

For patients with CP and branchial limb hypertonia where QoL, and not gain-of-function, is the desired outcome, the non-selective cervical VDR has the potential to address the shortcomings of other neurosurgical interventions such as SDR or ITBP. For patients with elements of dystonia, an SDR may exacerbate dystonic features [23]. For these patients ITBP is typically used, but it is known to have a higher complication rate than SDR, and typical lumbosacral ITBP may have reduced effect in the upper extremities compared with the lower extremities [24–27]. ITBP demonstrates reduction in upper extremity mAS scores by 0.8 to 1.8 compared with 1.75 to 2.5 in our cohort [26, 27]. Moreover, ITBP is relatively contraindicated in patient populations with low weight, epilepsy, scoliosis, and age less than four, and cervical VDR is not similarly constrained [28, 29]. This study included patients aged 7–34, and there was no cognitive threshold imposed. Due to the palliative nature of VDR, extensive rehabilitation to improve function is not required, and therefore this procedure does not require age or cognitive thresholds distinguishing it from other neurosurgical therapies.

Scoliosis has a high comorbidity in the CP population especially among those with GMFCS IV-V [30]. Scoliosis prevalence in our cohort was 67% (4/6), and all were patients with a GMFCS V. Progressive spinal deformity including kyphoscoliosis has been demonstrated in SDR [31, 32]. In the limited literature using cervical VDR, there has been no change in spinal deformity rate, and limiting the spinal opening is good practice to reduce this risk. No progressive spinal deformity was observed during the three-month follow-up. Ongoing follow-up is essential to substantiate the durability of these results.

Cervicothoracic VDR is compatible with additional therapeutic approaches; three patients (50%) underwent concurrent lumbosacral VDR. Cervicothoracic VDR can be combined with ITB catheter revision or lumbosacral VDR in the same operative experience.

Two patients with pre-existing respiratory conditions required transient Bi-pap, but there were no other complications. This suggests that extra care and monitoring postoperatively may be required in patients with pre-existing pulmonary disease.

### Gain of function surgery

While non-selective cervicothoracic VDR is not intended as a gain-of-function surgery, two cases presenting with isolated unilateral brachial hypertonia and predominantly distal symptoms showed notable functional improvement post-operatively. This suggests that the possibility of functional improvement should not be dismissed in select cases, although there is currently no predictive algorithm to guide us.

These surgeries were conducted on adults with fully realized developmental capabilities. The procedures were necessitated by their challenges in accessing appropriate care within the adult healthcare system. Transitional care inadequacies represent major barriers for CP patients [33]. In such cases with specialized procedures, pediatric neurosurgeons need to extend their expertise to the unique requirements of adult patients, ensuring that they receive comprehensive and effective care.

### Limitations

The current study has several potential limitations. It is a retrospective single institution series with heterogenous postoperative course and clinical testing. Patient demographics, type of hypertonia, and etiology varied. Additionally, the lack of a control group makes it difficult to prove efficacy. Long-term follow-up is not available for most patients. Larger multicenter studies with more patients and longer follow-up are necessary to further determine safety along with long-term functional benefits in these patients.

## Conclusions

Cervicothoracic VDR is safe and effective in the short-term and can be considered to treat severe upper limb hypertonia. Cervicothoracic VDR can provide quality of life improvements and symptomatic relief in patients with medically refractory upper limb hypertonia. Cervicothoracic VDR is appropriate when ITB is impractical; it can be considered a first-line surgical option in medically refractory brachial hypertonia patients with CP.

## Data Availability

No datasets were generated or analysed during the current study.

## Abbreviations

VDR: Ventral-dorsal rhizotomy
mAS: Modified Ashworth scale
CP: Cerebral palsy
CNS: Central nervous system
DBS: Deep brain stimulation
ITBP: Intrathecal baclofen pump
SDR: Selective dorsal rhizotomy
GMFCS: Gross motor function classification scale
EMG: Electromyography
BiPap: Bilevel positive airway pressure
